# The Protective Role of Bioactive Quinones in Stress-induced Senescence Phenotype of Endothelial Cells Exposed to Cigarette Smoke Extract

**DOI:** 10.3390/antiox9101008

**Published:** 2020-10-16

**Authors:** Ilenia Cirilli, Patrick Orlando, Fabio Marcheggiani, Phiwayinkosi V. Dludla, Sonia Silvestri, Elisabetta Damiani, Luca Tiano

**Affiliations:** 1 Department of Life and Environmental Sciences, Polytechnic University of Marche, 60121 Ancona, Italy; i.cirilli@pm.univpm.it (I.C.); p.orlando@univpm.it (P.O.); f.marcheggiani@univpm.it (F.M.); phiwayinkosi.dludla@mrc.ac.za (P.V.D.); l.tiano@staff.univpm.it (L.T.); 2 Biomedical Research and Innovation Platform, South African Medical Research Council, Tygerberg 7505, South Africa

**Keywords:** endothelial dysfunction, cigarette smoke, aging, vitamin K, menaquinone, ubiquinol, oxidative stress, mitochondrial dysfunction

## Abstract

Endothelial dysfunction represents the initial stage in atherosclerotic lesion development which occurs physiologically during aging, but external factors like diet, sedentary lifestyle, smoking accelerate it. Since cigarette smoking promotes oxidative stress and cell damage, we developed an in vitro model of endothelial dysfunction using vascular cells exposed to chemicals present in cigarette smoke, to help elucidate the protective effects of anti-inflammatory and antioxidant agents, such as ubiquinol and vitamin K, that play a fundamental role in vascular health. Treatment of both young and senescent Human Umbilical Vein Endothelial Cells (HUVECs) for 24 h with cigarette smoke extract (CSE) decreased cellular viability, induced apoptosis via reactive oxygen species (ROS) imbalance and mitochondrial dysfunction and promoted an inflammatory response. Moreover, the senescence marker SA-β-galactosidase was observed in both young CSE-exposed and in senescent HUVECs suggesting that CSE exposure accelerates aging in endothelial cells. Supplementation with 10 µM ubiquinol and menaquinone-7 (MK7) counteracted oxidative stress and inflammation, resulting in improved viability, decreased apoptosis and reduced SA-β-galactosidase, but were ineffective against CSE-induced mitochondrial permeability transition pore opening. Other K vitamins tested like menaquinone-4 (MK4) and menaquinone-1 (K1) were less protective. In conclusion, CSE exposure was able to promote a stress-induced senescent phenotype in young endothelial cells likely contributing to endothelial dysfunction in vivo. Furthermore, the molecular changes encountered could be offset by ubiquinol and menaquinone-7 supplementation, the latter resulting the most bioactive K vitamin in counteracting CSE-induced damage.

## 1. Introduction

Over the past half century, a steady decline in deaths from cardiovascular diseases (CVDs) has been observed in highly developed countries, however, atherosclerosis-associated vascular diseases remain the leading cause of morbidity and mortality worldwide [1,2]. Atherosclerosis is a chronic inflammation of arteries and it represents an age-related disease that develops from endothelial dysfunction characterized by oxidative imbalance. The major modifiable risk factors for CVDs are diet, sedentary lifestyle, excessive alcohol consumption and tobacco smoke. The latter is a highly complex aerosol of >5000 compounds, including reactive oxygen species (ROS), reactive nitrogen species, carbon monoxide, nitric oxides, nicotine, polycyclic hydrocarbons, cadmium, as well as other metals and oxidants [3]. In particular, the oxidant fraction of cigarette smoke is thought to be a major trigger for the formation of atherosclerotic plaques and pathological thrombi [4]. ROS excess may increase levels of oxidized LDL (oxLDL) which is responsible for the progression of atherosclerosis and causes direct damage to endothelial cells promoting cellular apoptosis via lectin-type oxidized LDL receptor 1 (LOX-1) [5]. The anti-apoptotic protein Bcl-2 is down-regulated due to the activation of the apoptotic signaling proteins caspase-3 and caspase-9 [5] accompanied by an increase in mitochondrial permeability transition pore (mPTP) opening which releases cytochrome c that activates caspase-1. Moreover, the shift to a vascular pro-oxidative state and activation of NADPH oxidase significantly affects signaling pathways regulating vascular tone and endothelial cell proliferation, migration and differentiation [6], and may significantly contribute to inflammation. In fact, cigarette smoke-derived ROS trigger the activation of nuclear factor-κB (NF-κB) resulting in the expression of pro-inflammatory cytokines, such as interleukin-6 and interleukin-8, adhesion molecules, such as intercellular adhesion molecule 1 (ICAM-1) and vascular cell adhesion molecule 1 (VCAM-1) on the surface of endothelial cells [7]. Furthermore, in vitro studies have shown that cigarette smoke is able to induce premature senescence in different cell types [8,9,10] increasing the expression of p16^INK4a^, and consequently blocking the cell cycle [11]. Thus, therapeutic strategies to slow down this senescence-induced process is an attractive approach for improving vascular function and reducing the risk of developing vascular dysfunction.

In this context some quinones, such as coenzyme Q_10_ (CoQ_10_) and vitamin K, fundamental molecules in cardiovascular health, could play an important role in protecting endothelia from external stress. CoQ_10_ is an endogenous lipophilic cofactor present in humans that supports mitochondrial bioenergetics and its reduced form, ubiquinol (QH), is a potent antioxidant [12] in addition to being more bioavailable compared to the oxidized form, thanks to its more hydrophilic properties [13]. The antioxidant properties of coenzyme Q_10_ can protect endothelial cells by reacting directly with oxidant species and by potentiating the activity of antioxidant enzymes such as superoxide dismutase and catalase [14]. Moreover, CoQ_10_ also exerts anti-inflammatory properties via the reduction of NF-κB-dependent gene expression [15]. In fact, a negative correlation between CoQ_10_ and inflammatory markers (TNF-α and IL-6) was shown after 12 weeks of 300 mg/day CoQ_10_ supplementation in coronary artery disease patients [16]. Moreover, chronic inflammation is a peculiar feature of senescence characterized by the development of a specific senescence-associated secretory phenotype (SASP). In this respect, ubiquinol supplementation showed anti-aging potential in endothelial cells by counteracting the development of senescence-associated β-galactosidase positive cells and by suppressing the expression of SASP phenotype [17,18]. In addition, a decrease in endogenous CoQ_10_ content is known to occur in conditions associated with oxidative stress and mitochondrial dysfunction such as in degenerative pathologies as well as in physiological conditions during the aging process [19,20]. Consequently, under these conditions, CoQ_10_ supplementation and in particular its reduced form, could represent a beneficial integration strategy.

**Figure 1:**
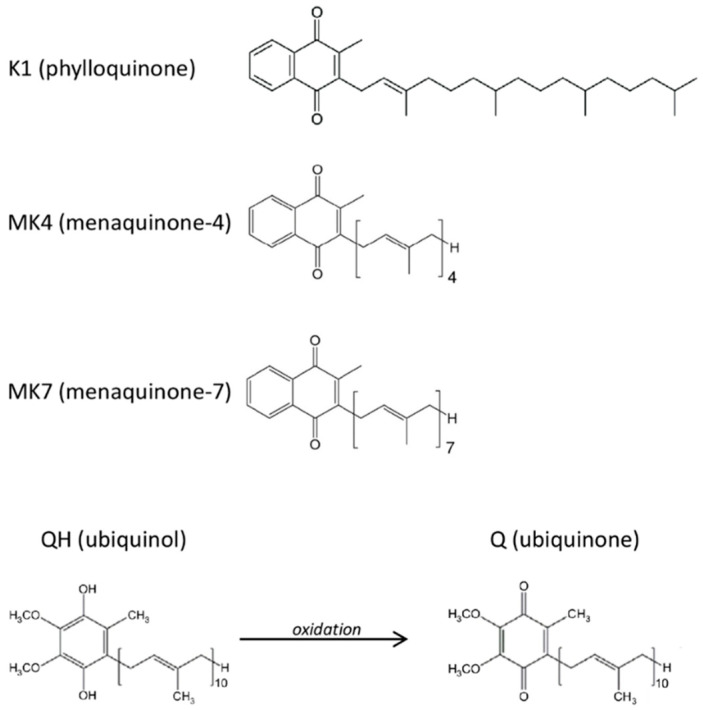
Molecular structures of the tested quinones.

Other biologically relevant quinones are vitamin K compounds. These molecules constitute a family of fat-soluble vitamins comprising structurally similar molecules including two main natural forms: phylloquinone (vitamin K1) and menaquinones (collectively known as vitamin K2). The latter are a series of vitamins which differ in isoprenoid side chain and the most common are menaquinone-4 (MK-4) and menaquinone-7 (MK-7) (Figure 1). The principal known function of vitamin K is as a cofactor for γ-carboxyglutamyl carboxylase (GGCX) required for the post-translational carboxylation of vitamin K-dependent proteins (VKDPs), i.e., it catalyzes a reaction in which specific glutamate residues (Glu) found in VKDPs, are converted to γ-carboxyglutamate (Gla). The VKDPs thus activated can perform multiple functions, some of which remain still unclear, but are suspected to play roles in different processes such as bone and cardiovascular mineralization, vascular hemostasis, energy metabolism, immune response, brain metabolism and in cellular growth, survival, and signaling [21]. In addition, vitamin K appears to suppress the pro-inflammatory cytokines production through a non-carboxylative pathway, by modulating the gene expression of pro-inflammatory markers [22]. Thus, vitamin K could play a fundamental role in the protection of the endothelium under stress conditions, however the different capacity of the K vitamins and the mechanisms of action have not yet been completely explored.

It is known that ubiquinol and vitamin K, through different modes of action, play a fundamental role in vascular health. While for ubiquinol a large body of evidence supports its bioenergetic but also antioxidant and anti-inflammatory activities, the role of vitamin K, mainly in its menaquinone form (K2), has been limited to its ability to prevent vascular calcification. Therefore, to gain further information on the role of quinones in vascular health, we first developed an in vitro model of endothelial dysfunction using vascular cells exposed to chemicals present in cigarette smoke known to promote oxidative stress and cell damage. This model was then used to compare the effects of anti-inflammatory and antioxidant agents, such as ubiquinol and the major K vitamins (K1, MK4, MK7), focusing on the prevention of oxidative damage and premature senescence.

## 2. Materials and Methods 

### 2.1. Cellular Model

Primary human umbilical vein endothelial cells (HUVECs) were purchased from Clonetics (Lonza, Basel, Switzerland). The cells were grown in Endothelial Cell Growth Medium-2 (EGM-2) (Lonza) and maintained at 37 °C in a humidified atmosphere and 5% CO_2_. Young cells from passages 5–9 were used for the treatments, while cells from passages 18–20 were used as senescent control. Cells were seeded in 24-well plates or in 6-well plates at an optimal density of 6 × 10^3^ cells/cm^2^ and treated after 48 h. To establish cigarette smoke extract (CSE) effects, the cells were first cultured with CSE in the range of 0.106 puffs/mL up to 1.7 puffs/mL for 24 h and the dose-dependent changes in relation to viability decrease and ROS production were monitored. The 24 h exposure time was defined according to Lee et al. experimental design [23]. Subsequently, a sub-lethal concentration of CSE (0.425 puffs/mL) was used to investigate its effect on young HUVECs in terms of oxidative stress, inflammation and senescence markers. After verifying in HUVECs whether CSE influences quinones bioavailability after 24 h exposure, the role of ubiquinol and vitamin K supplementation in counteracting CSE-induced damage was evaluated. Young HUVECs were supplemented for 24 h with ubiquinol (kindly donated by Kaneka, Tokyo, Japan) or K vitamins: K1 (purchased from Sigma-Aldrich, St. Louis, MO, USA), MK4 and MK7 (kindly donated by Gnosis-Lesaffre, Milan, Italy) (Figure 1) solubilized in water using a mixture of glycerol and the emulsifying agent PEG 60-hydrogenated castor oil (Cremophor; BASF SE Chemical Co., Ludwigshafen, Germany) (QH: glycerol: HCO60 = 0.4: 0.6: 1). The cell culture medium was then replaced with 0.425 puffs/mL CSE medium containing the same compounds and incubated for a further 24 h (cell viability, cellular and mitochondrial ROS, mPTP and caspase-1 analysis) or 48 h (SA-β-galactosidase).

### 2.2. Cigarette Smoke Extract Preparation

Cigarette smoke extract (CSE) was prepared by a modification of the method of Carp and Janoff [24] using a commercially available filter-tipped cigarette composed of: tar (10 mg/cigarette), carbon monoxide (10 mg/cigarette), nicotine (0.8 mg/cigarette). Briefly, CSE sampling was performed using a modified syringe-driven apparatus in which a cigarette was aspirated with a 20 mL syringe which was filled in 2 s. Subsequently, by using a T-junction, the smoke was bubbled through EGM-2 cell culture medium for 10 s. Each cigarette was consumed in 17 aspirations (puffs) and the smoke from 10 cigarettes was bubbled into 50 mL of culture medium, thus the stock solution concentration was 3.4 puffs/mL. The resulting medium was adjusted to pH 7.4 and sterilized by filtration through a 0.20 µm pore filter, aliquoted and stored at −20 °C. For the experiments, the stock solution was diluted in the cell culture medium. The reproducibility and stability of CSE was verified by comparing the effect of fresh and stored CSE extract over a 6-month period and toxicity data were consistent (data not shown).

### 2.3. Quinones Bioavailability

Bioavailability of quinones was evaluated in young Human Umbilical Vein Endothelial Cells (HUVECs) incubated with 10 µM of ubiquinol or vitamin K in the presence or absence of 0.425 puffs/mL CSE for 24 h. After treatment, the cells were washed, detached, counted, resuspended in 50 μL of PBS and extracted with 250 μL of propanol or ethanol for total CoQ_10_ and its oxidative status as well as K vitamins analyses, respectively. After vigorous vortexing, the extraction mixture was centrifuged at 20,900 *g*/4 °C/2 min and 50 μL of supernatant were analyzed by high-performance liquid chromatography (HPLC).

CoQ_10_ was quantified by electro-chemical detection (ECD; Shiseido, Tokyo, Japan) where both reduced and oxidized forms can be detected due to a post-separation reducing column (Shiseido) able to fully reduce the eluted fractions as previously reported [25]. K vitamins levels were assayed in HUVECs suspensions using an HPLC system (9300, YL Instrument, Anyang, Republic of Korea) equipped with a fluorescence detector (Nanospace SI-2; Shiseido) and an analytical column (2.6 µm C18 100A, 100 × 4.6 mm; Phenomenex Kinetex, Torrance, CA, USA) connected to a post-chromatographic reducing column (CQ-R 2.0 × 20 mm; Shiseido). The mobile phase used was ethanol:water (97:3, *v/v*) and the flow rate was adjusted to 0.7 mL/min. The optimized detection wavelengths were 335 nm (excitation) and 430 nm (emission). Under these conditions the detection was 13 min long and it was possible to separate K1, MK4 and MK7. External standards (purchased from Sigma-Aldrich) were used to quantify K vitamins concentrations. Representative chromatograms of the K vitamins and CoQ10 are reported in Figure S1. Intracellular total CoQ_10_ and K vitamins levels are expressed as nmol/10^6^ cells, while the oxidative status of CoQ_10_ is reported as the percentage of oxidized CoQ_10_/total CoQ_10_.

### 2.4. Viability, Cellular and Mitochondrial ROS Content and mPTP Assay

Cell viability, total cellular and mitochondrial ROS levels and mitochondrial Permeability transition pore (mPTP) opening were evaluated as previously described [26,27]. Flow cytometry coupled with appropriate fluorescent probes was conducted on a Guava EasyCyte flow cytometer equipped with GuavaSoft 2.7 software and an excitation source at 488 nm (Merck Millipore, Darmstadt, Germany). Emission fluorescence intensities were recorded in different channels on an average of 5000 cells from each sample.

Briefly, viability and intracellular ROS levels analysis were conducted simultaneously using 10 µM of the ROS-sensitive probe carboxy-2,7-dichlorofluorescein diacetate (carboxy-H_2_DCFDA) (Invitrogen) and Guava ViaCount (Merck Millipore), a fluorescent stain formulation that discriminates live, dead and apoptotic cells on the base of differential permeabilities of two DNA-binding dyes. For data analysis, a “high” ROS region was arbitrarily defined in the green channel, while live, apoptotic and dead cells gates were defined in the dot plot produced from the analysis of yellow and red fluorescence channels, using the fluorescence distribution of CSE-treated HUVECs as a reference. The same gates were used for the analysis in all subsequent experiments.

MitoSOX™ Red (Invitrogen, Carlsbad, CA, USA) and MitoProbe^TM^ Transition Pore Assay Kit (Invitrogen) were used to evaluate mitochondrial ROS and mPTP opening, respectively. For quantitative analysis of fluorescence distribution, gate defining regions with “high” ROS and “high” mPTP opening were arbitrarily set using untreated and CSE-treated cells as a reference. The same settings were then maintained for all subsequent experiments. Representative flow cytometric graphs of the gate defining regions are reported in Figure S2A–C.

### 2.5. Activated Caspase-1 Detection

The levels of caspase-1 were evaluated using FAM-fluorochrome inhibitor of caspases (FAM-FLICA) conjugated probes (ImmunoChemistry Technologies, Bloomington, MN, USA). FLICA is cell permeant and covalently binds to active caspase enzymes. Briefly, after treatment the cells were detached, washed and stained with 1× FLICA solution in the dark for 50 min at 37 °C. Subsequently, cells were counterstained with propidium iodide and analyzed immediately by flow cytometry. The fluorescence results from the two channels were analyzed together and four regions were identified: live, negative or positive caspase cells and dead, negative or positive caspase cells. Representative flow cytometric dot plot is reported in Figure S2D.

### 2.6. Senescence-associated β-galactosidase Staining

Senescence-Associated β-galactosidase activity (SA-β-gal), a common molecular marker of cellular aging, was quantified using a Senescence Detection Kit (Abcam, Cambridge, UK). After treatment, non-confluent HUVECs cultured in 24-well plates were fixed for 15 min at room temperature, then washed twice in PBS and incubated overnight at 37 °C with Staining Solution Mix (containing the X-Gal substrate). SA-β-Gal was assessed under light microscopy at 200× magnification. The percentage of positive cells was determined by counting at least 500 cells/sample.

### 2.7. Data Analysis

Each experiment was performed at least three times in different experimental sessions. Data were analyzed using GraphPad Prism 6 (GraphPad Software, La Jolla, CA, USA) and one-way Analysis of Variance (ANOVA) followed by Tukey’s multiple comparisons test was used to determine statistical significance. *p* value ≤ 0.05 was considered statistically significant and represented as: “*” *p* ≤ 0.05, “**” *p* ≤ 0.01 or “***” *p* ≤ 0.001. Data are represented as mean ± SD.

## 3. Results

### 3.1. CSE Altered Oxidative Status, Mitochondrial Health, Pro-Inflammatory Caspase-1 Activity and Promoted Cellular Senescence

Cigarette smoke, one of the main risk factors for cardiovascular diseases, creates a strongly oxidizing environment which causes important changes at the cellular level and promotes premature aging. In particular, endothelial damage represents an early manifestation of atherosclerotic lesions and plays a prominent role in its development.

**Figure 2:**
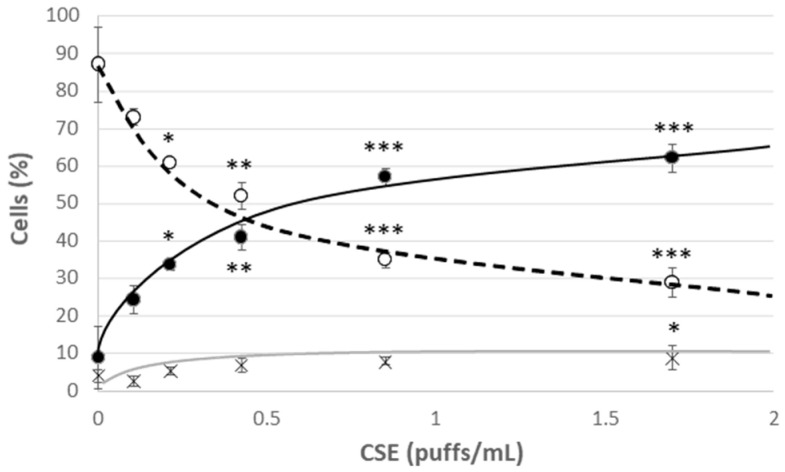
Cell viability after 24 h treatment of young HUVECs with increasing doses of CSE. O Live cells, ● Apoptotic cells, X Dead cells. Significance was calculated with respect to young untreated cells (0 puffs/mL CSE). * *p* ≤ 0.05, ** *p* ≤ 0.01, *** *p* ≤ 0.001.

**Figure 3:**
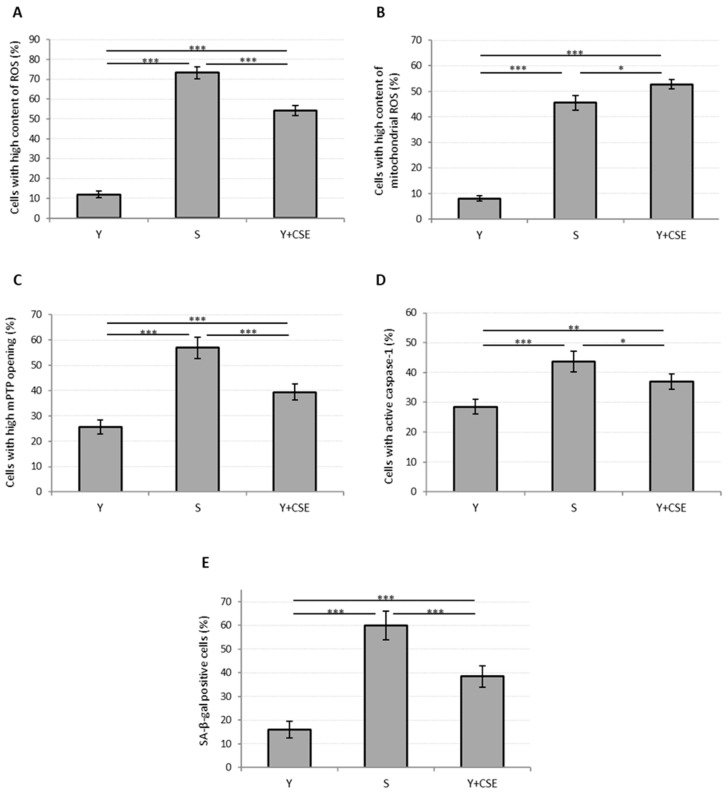
Oxidative stress, mitochondrial functionality, caspase-1 activation and senescence in young HUVECs exposed to CSE (0.425 puffs/mL for 24 h). The histograms depict the results obtained from untreated young (**Y**) and untreated senescent (**S**) HUVECs and young HUVECs exposed to CSE (Y + CSE), that show the percentage of cells with: (**A**) high ROS content, (**B**) high mitochondrial ROS content, (**C**) high mPTP opening, (**D**) active caspase-1, (**E**) SA-β-galactosidase positive cells. * *p* ≤ 0.05, ** *p* ≤ 0.01, *** *p* ≤ 0.001.

Young HUVECs were first exposed to a range of CSE (0.106 puffs/mL–1.7 puffs/mL) for 24 h in order to develop a model of CSE-induced cellular stress and premature aging. A strong decrease in viability was observed already in cells incubated with the lowest CSE concentration, which however did not lead to a significant increase in the mortality rate, but rather to a significant increase in the percentage of apoptotic cells (Figure 2). This effect was associated with the observed increase in the mPTP opening compatible with the early apoptotic phases (Figure 3C). In addition, at all the tested CSE concentrations, a dose-dependent and highly significant increase in ROS production was observed (Figure S3). In particular, a sub-lethal CSE concentration (0.425 puffs/mL) significantly increased the percentage of cells showing a high cytosolic ROS content from 12 ± 2% in the unexposed young control cells to 54 ± 3% in CSE-exposed young cells (*p* ≤ 0.001) (Figure 3A). Mitochondrial ROS content, mainly represented by superoxide anion (O_2_^•−^), the major reactive oxygen species deriving from mitochondrial metabolism, also increased significantly following CSE treatment (Y: 8 ± 1%; Y + CSE: 53 ± 2%; *p* ≤ 0.001), to levels even higher than those observed in senescent HUVECs (S: 46 ± 3%; *p*_Y + CSE-S_ ≤ 0.05) (Figure 3B). This suggests that CSE has a clear mitochondrial toxicity.

Parallel to the increase in mitochondrial ROS, a significant opening in mPTP was observed (Y: 26 ± 3%; Y + CSE: 39 ± 3%; *p* ≤ 0.001) (Figure 3C), suggesting that incubation of young HUVECs in the presence of CSE can trigger mitochondrial dysfunction. Moreover, an increased activation of caspase-1 (Y: 29 ± 2%; Y + CSE: 37 ± 3%; *p* ≤ 0.01), a relevant marker of inflammation and apoptosis, was observed (Figure 3D).

In summary, exposure of young cells to CSE caused an increase in both oxidative stress and apoptosis/inflammation biomarkers, although these indices, with the exception of mitochondrial ROS, were significantly lower than those observed in senescent cells. The same pattern was also observed analyzing the activity of SA-β-galactosidase, a specific senescence marker (Y: 16 ± 4%; Y + CSE: 39 ± 5%; *p* ≤ 0.001) (Figure 3E). These data suggest that exposure of young endothelial cells to 0.425 puffs/mL CSE was able to induce biochemical changes similar to those that occur in replicative senescence.

### 3.2. CSE Did not Affect Exogenous Quinones Bioavailability and CoQ_10_ Oxidative Status

Subsequently, we verified whether CSE could affect the absorption of quinones and CoQ_10_ oxidative status. Young HUVECs were incubated for 24 h with 10 μM quinones in the standard culture medium or in the same medium containing 0.425 puffs/mL CSE. Untreated HUVECs were used as a control and the endogenous levels of the K vitamins in these were undetectable, whereas total CoQ_10_ level was 0.014 ± 0.001 pmol/10^6^ cells where approximately 50% was in the oxidized form. Notably, CSE treatment did not influence the levels of endogenous CoQ_10_ content nor its oxidative status.

**Figure 4:**
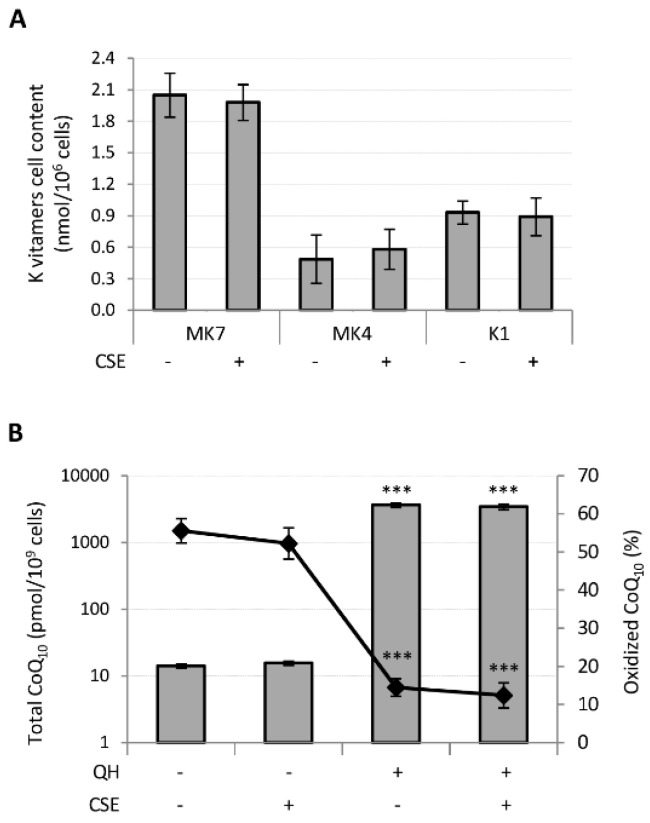
Effect of CSE exposure (0.425 puffs/mL for 24 h) on quinones bioavailability and CoQ_10_ oxidative status in young HUVECs. K vitamin cellular content (**A**) and CoQ_10_ cellular content and oxidative status (**B**), were evaluated in young HUVECs treated with 10 µM of quinones alone or in association with CSE exposure. + and − represent exposure or not exposure, respectively, to the indicated substances. Untreated and CSE exposed cells were used as controls. *** *p* ≤ 0.001 vs. untreated cells.

Following supplementation, MK7 was absorbed with greater efficiency than the other K vitamins since its cellular content resulted 4 and 2 times greater than MK4 and K1, respectively, whereas MK4 showed the lowest bioavailability. CSE had no effect on their bioavailability (Figure 4A).

Similarly, CSE exposure did not affect the cellular content nor the oxidative status of CoQ_10_ in QH supplemented cells (Figure 4B). However, incubation of HUVECs in the presence of 10 μM QH resulted in a 250-fold increase in total CoQ_10_ and a remarkable decrease in the percentage of oxidized CoQ_10_ from 56 ± 3% to 14 ± 2% (*p* ≤ 0.001) (Figure 4B).

### 3.3. Quinones Differentially Protect against CSE-Induced Cytotoxicity, Oxidative Stress, Mitochondrial Dysfunction, Pro-Inflammatory Caspase-1 Activation and Cellular Senesence

**Figure 5:**
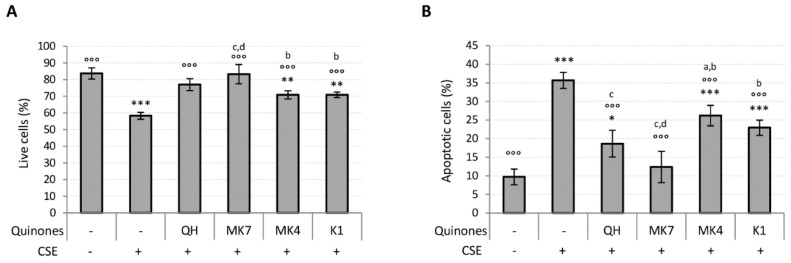
Effect of quinones supplementation on viability loss caused by CSE exposure (0.425 puffs/mL for 24 h) in young HUVECs. Cells were supplemented with 10 µM quinones for 24 h followed by replacement with CSE medium containing the same quinones and incubated for a further 24 h. (**A**) Percentage of live cells after treatment, (**B**) percentage of apoptotic cells after treatment. + and – represent exposure or not exposure, respectively, to the indicated substances. °°° *p* ≤ 0.001 vs. CSE-treated cells; * *p* ≤ 0.05, ** *p* ≤ 0.01, *** *p* ≤ 0.001 vs. untreated young HUVECs; a *vs* QH; b *vs* MK7; c *vs* MK4; d *vs* K1.

Ubiquinol and K vitamin supplementation (10 μM) efficiently counteracted the loss of viability and apoptosis associated with CSE exposure (Figure 5A,B). However, MK7 showed greater protective effects even at the lowest tested concentration (1 μM) (Figure S4A,B). The results also show that QH and MK7 were the most effective molecules for limiting CSE-induced loss in viability (Figure 5A). Furthermore, MK7 was the only vitamer able to completely counteract CSE-induced apoptosis whereas MK4 and K1 were not as effective (Figure 5B).

**Figure 6:**
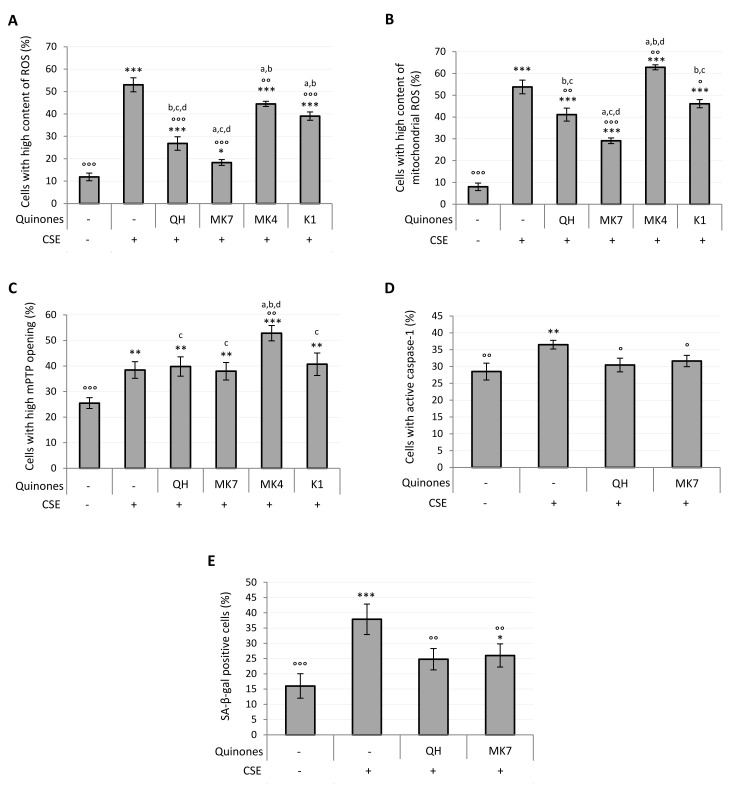
Effect of quinones supplementation on oxidative stress, mitochondrial functionality, caspase-1 activation and senescence in young HUVECs exposed to CSE (0.425 puffs/mL for 24 h). Cells were supplemented with 10 μM quinones followed by replacement with CSE medium containing the same quinones and incubated for a further 24 h (except in the case of SA-β-galactosidase activity where cells were incubated for 48 h). Percentage of cells with: (**A**) high ROS content, (**B**) high mitochondrial ROS content, (**C**) high mPTP opening, (**D**) active caspase-1, (**E**) SA-β-galactosidase positive cells. + and – represent exposure or not exposure, respectively, to the indicated substances. ° *p* ≤ 0.05, °° *p* ≤ 0.01, °°° *p* ≤ 0.001 vs. CSE-treated cells; * *p* ≤ 0.05, ** *p* ≤ 0.01, *** *p* ≤ 0.001 vs. untreated HUVECs; a *vs* QH; b *vs* MK7; c *vs* MK4; d *vs* K1.

Parallel to the increase in viability observed in quinone-supplemented cells exposed to CSE, a dose-dependent decrease in the percentage of cells characterized by a high cytosolic and mitochondrial ROS content was observed (Figure S4C,D). Figure 6A shows that at 10 μM all quinones significantly decreased intracellular ROS content (QH, MK7, K1: *p* ≤ 0.001; MK4: *p* ≤ 0.01), although none were able to completely prevent the CSE-induced ROS increase. Notably, QH and MK7 were both significantly effective even at the lowest concentration tested (1 μM) (Figure S4C). With the exception of MK4, all quinones decreased mitochondrial ROS production resulting from CSE exposure (Figure 6B), with MK7 being the most successful (CSE: 54 ± 5%; 10 µM MK7 + CSE: 29 ± 3%, *p* ≤ 0.001; QH + CSE: 41 ± 3%; *p* ≤ 0.01; K1 + CSE: 46 ± 3%, *p* ≤ 0.05). Its efficacy was also observed even at the lowest concentration tested (1 μM) (Figure S4D). As shown in Figure 6B, MK4 significantly promoted the formation of mitochondrial superoxide anion, which could be associated with the exacerbation of mPTP opening observed in the presence of this compound as reported in Figure 6C (CSE: 38 ± 3%; MK4 + CSE: 53 ± 3%; *p* ≤ 0.01). All the other quinones, despite being protective in relation to mitochondrial generation of ROS, were unable to reduce mPTP opening induced by CSE exposure (Figure 6C).

CSE pro-inflammatory effect in young HUVECs, likely associated with the pro-oxidant effect observed, was characterized by an increased activity of caspase-1. Since the results reported in Figure 6A,B showed that QH and MK7 were the most effective quinones in the prevention of CSE-induced oxidative stress, we chose to test the activity of just these two quinones toward the inhibition of caspase-1 activation. As shown in Figure 6D, supplementation with 10 μM QH or MK7 was able to completely prevent the pro-inflammatory effect resulting from CSE exposure (CSE: 37 ± 1%; QH + CSE: 30 ± 2%; MK7 + CSE: 32 ± 2%; *p* ≤ 0.05). Indeed, the percentage of activated caspase-1 cells exposed to these quinones were comparable with those of the negative control cells (Ctrl: 29 ± 3%).

Oxidative stress, mitochondrial dysfunction and chronic inflammation are characteristic features of the senescent phenotype. Accordingly, CSE exposure in the proposed experimental setting was able to induce SA-β-galactosidase (SA-β-gal), a specific marker of cellular aging. From the results shown in Figure 6A,B,D, we again chose to test the two most effective quinones, QH and MK7 against prevention of the CSE-induced senescence phenotype. Indeed, the CSE-induced increase in the percentage of SA-β-gal positive cells (CSE: 38 ± 5%) was found to be effectively prevented by supplementation with 10 μM QH (QH + CSE: 25 ± 3%; *p* ≤ 0.01) and 10 μM MK7 (MK7 + CSE: 26 ± 4%; *p* ≤ 0.01) (Figure 6E).

## 4. Discussion

The vascular endothelium plays a pivotal role in cardiovascular function and its dysfunction is an early manifestation of atherosclerotic disease. In the present study, cigarette smoke exposure was used as a model of endothelial insult and its cytotoxicity was used to identify the beneficial activity of potential protective molecules. In particular, young endothelial cells (HUVECs) were used to investigate the toxicity and the pro-senescence effects of cigarette smoke extract (CSE) comparing CSE-exposed young cells with untreated young and senescent cells.

CSE exposure in dermal and lung fibroblasts has previously been reported to reduce viability in a dose- and time-dependent manner [8,28]. In addition, it has been demonstrated that CSE exposure increases phosphatidylserine residues externalization [28] and caspase 3/7 activity [29], characteristic features of early phases of apoptosis in lung fibroblasts and endothelial cells. In agreement with these data, we found a significant decrease in young HUVECs viability already at the lowest tested CSE concentration (0.106 puffs/mL) after 24 h incubation. Decrease in the percentage of live cells was not associated with a parallel increase in the percentage of dead cells, but rather with a significant increase in apoptotic-like cells. On the contrary, Csordas et al. suggested that protein damage caused by CSE activates autophagy, ultimately leading to necrotic death of HUVECs [30]. In this context, the pathways leading to CSE-induced cell death could be influenced by the CSE concentration used as reported in the literature: lower concentrations induce caspase-independent apoptosis-like programmed cell death through the recruitment of the Apoptosis Inducing Factor (AIF); whereas incubation with higher concentrations interrupts apoptotic signaling and induces necrosis because cell damage becomes too extensive [31]. Apoptosis may occur in response to specific stimuli such as heat stress, radiation, steroids, and oxidative stress. Chemicals contained in cigarette smoke are known to trigger an imbalance between ROS production and antioxidant system resulting in oxidative stress [32]. There are evidences that CSE exposure causes mitochondrial impairment associated with loss of cellular ATP, rapid depolarization of mitochondrial membrane potential and activation of permeability transition pore followed by apoptotic cell death [31,33]. These data support the hypothesis that cigarette smoke may contribute to increasing the risk of cardiovascular disease also by promoting mitochondrial dysfunction and damage. In fact, in our model, CSE treatment of young HUVECs produced a significant increase in ROS content both in the cytosol and mitochondria, in addition to an increase in mPTP opening. Increased levels of mitochondrial ROS, mainly consisting in O_2_^•−^, are implicated in pro-atherogenic vascular phenotypic alterations, including expression of pro-inflammatory genes [34]. In particular, exposure to CSE of human coronary arterial endothelial cells was shown to induce NF-κB activation which promotes the transcription of a large range of genes implicated in inflammation, including cytokines (IL-6, IL-1β and TNFα), chemokines and adhesion molecules [7]. In particular, IL-1β is activated by active caspase-1. Pro-caspase-1 can itself be activated by the inflammasome, a multiprotein platform activated upon non-microbial and stress-associated signals, including ROS and extracellular adenosine triphosphate [35]. Our results confirm that CSE exposure can lead to inflammation by increasing caspase-1 activation in young HUVECs.

An imbalance between ROS production and antioxidant defenses resulting in oxidative stress that promotes mitochondrial dysfunction and inflammation, is also observed in senescent cells. The analogy in the present study between CSE-exposed young HUVECs and senescent HUVECs is confirmed by the levels of SA-β-galactosidase observed, which increases following exposure of young cells to 0.425 puffs/mL CSE for 48 h. On the other hand, SA-β-galactosidase activation may also reflect lysosomal enrichment as a consequence of mitophagy to remove the impaired mitochondria [36].

Overall, the data obtained on our experimental model of CSE-exposed endothelial cells, show that in addition to a consistent induction of oxidative imbalance, followed by an increased inflammatory state and altered mitochondrial function, CSE exposure was also able to induce a premature senescence. All these changes are critical to the development of endothelial dysfunction and may promote the onset and the progression of atherosclerotic lesions.

Growing evidences indicate that CSE-induced oxidative stress and inflammation may be prevented by antioxidant molecules, such as alpha-tocopherol (vitamin E) and ascorbic acid (vitamin C) [37], resveratrol [29], glutathione, melatonin, lipoic acid and Coenzyme Q_10_ (CoQ_10_) [38]. Ubiquinol (QH), the active form of CoQ_10_, and vitamin K play a fundamental role in vascular health in physiological conditions by protecting the endothelium from oxidative damage, inflammation and by ensuring the functionality of important vitamin K-dependent proteins (VKDP), mainly known so far for their functions in preventing vascular calcification (MG- and GLA-rich proteins) [39] although VKDP have also recently been associated with anti-inflammatory activities [40]. Hence, it is possible that QH and K vitamins could also counteract CSE-induced damage in endothelial cells. Kaisar et al. found that 2 h of 10 µg/mL oxidized CoQ_10_ supplementation followed by CSE treatment (12 h) was able to reduce the release of pro-inflammatory cytokines (IL-6, IL-8), whereas it weakly prevented the up-regulation of PECAM-1, VCAM-1 and E-selectin [38]. On the contrary, Gairola et al. showed that CoQ_10_ supplementation in apoE-deficient mice was not able to counteract CSE-induced atherosclerotic lesions progression [41]. Nevertheless, there is not much data in the literature regarding the effect of CoQ_10_ supplementation in response to oxidative stress induced by cigarette smoke. However, some studies suggested the protective role of CoQ_10_ supplementation in both in vitro H_2_O_2_-treated HUVECs [18] and in vivo models: humans with coronary artery disease [16] and in senescence-accelerated mice [42]. On the contrary, research on the role of K vitamins in aging and in stress conditions has been largely neglected.

The tested quinones share a similar chemical structure, however their biological function has significantly differentiated during evolution. While their basic chemical function as electron and proton carriers underlies their activities, their cellular location and their interaction with the complex biochemical network has significantly evolved from prokaryotes to eukaryotes and within metazoans [43]. Although there is an advanced knowledge of ubiquinone biochemistry and its role as lipophilic antioxidant and in mitochondria bioenergetics, knowledge of K vitamins has been mainly limited to their role as cofactors of carboxylase, especially as activators of 19 vitamin K-dependent proteins, most of them related to coagulative function or implicated in bone and cardiovascular health as calcium-binding proteins. Nonetheless, in the last decade research has started to consider other biological functions in relation to their antioxidant, anti-inflammatory activities and as regulators of the cell cycle [44,45], that could derive from chemical features shared by other biological quinones including Coenzyme Q10. In particular, Vos et al. have shown that menaquinones are involved in mitochondrial activity and in rescuing PINK1 deficiency in a drosophila model [46].

In the present study we found a strong protective effect of both QH and MK7 supplementation as both quinones were able to reduce viability loss, oxidative stress and inflammation promoted by CSE exposure in young HUVECs, as well as preventing SA-β-gal increase, thus slowing down cell aging. In particular, at 10 µM these molecules improved oxidative stress (reducing cytosolic and mitochondrial ROS) but they were unable to prevent mitochondrial dysfunction characterized by increased mPTP opening. Indeed, CSE mitochondrial toxicity and mPTP opening might be triggered indirectly by alterations in mitochondrial membrane potential. In this respect, Yang and Liu. reported that CSE exposure decreases the expression of the anti-apoptotic protein Bcl-2 and concomitantly induces the proapoptotic protein Bax [47]. These proteins regulate key steps in the apoptotic pathway by modulating mitochondrial membrane potential (MMP) [48]. Moreover, MMP is also affected by CSE exposure following the activation of adenine nucleotide translocator (ANT) with consequent uncoupling effects [49]. Caspase-1 is also a pro-apoptotic protein [50] and both QH and MK7 were able to inhibit its activation induced by CSE exposure in HUVECs. Intriguingly, in relation to viability and oxidative status of CSE-treated cells, MK7 was the most active molecule among the quinones tested and its anti-apoptotic and mitochondrial ROS curbing activity was already highly significant at 1 μM concentrations, 10 times lower than the effective dose of QH.

The suppression of oxidative stress could be the main beneficial function of QH and MK7 in our experimental system, leading to general improvement in cellular functionality. This effect may not necessarily be associated with the direct radical scavenging role of these molecules. In fact, a recent in vitro study showed that Nrf-2 (nuclear factor erythroid 2-related factor 2), the main mediator of cellular adaption to oxidative stress, and its target genes, were strongly increased by CSE in a dose-dependent manner [51,52]. Following oxidative insult, Nrf-2 dislodges from Kelch-like erythroid cell-derived protein 1 (Keap-1) binding domain and translocates into the nucleus where it binds to the corresponding antioxidant response element (ARE). This ultimately leads to overexpression of detoxifying agents like heme oxygenase (decycling) 1 (HMOX1) and NAD(P)H quinone dehydrogenase 1 (NQO1). An elevated level of NQO1 is an indicator of the activation of cellular defense mechanisms in response to oxidative stimuli such as CSE exposure. However, Fratta Pasini et al. found that in HUVECs exposed to smokers’ serum, the expression of Nrf-2, HMOX1 and of glutamate-cysteine ligase catalytic (GCLC) subunit, the enzyme that catalyzes the rate-limiting step of GSH synthesis, were decreased [53] implicating a reduction in antioxidant defenses. Antioxidants capable of acting intracellularly by stimulating ARE pathways are able to promote the antioxidant response, therefore in this scenario, antioxidants supplementation could help in counteracting oxidative damage caused by CSE. In particular, the great efficiency of vitamin K in preventing the damage caused by smoking as highlighted in our study, could be mediated by NQO1 whose expression is known to increase after oxidative stimuli [52] and this in turn could lead to greater vitamin K recycling efficiency making it more available for carboxylases responsible for VKDPs activation.

In the present study, MK7 was surprisingly the most effective molecule, also in comparison to ubiquinol that is well known for its antioxidant activity and protective role in the vascular system. The other K vitamins tested, MK4 and K1, showed only a slight improvement in viability and cytosolic ROS content. On the contrary, MK4 amplified the CSE-induced increase in mitochondrial ROS and exacerbated mPTP opening, promoting mitochondrial dysfunction. Mitochondrial perturbation by MK4 and the lower bioavailability of K1 and MK4 could underlie their reduced efficacy in the proposed cellular stress model. In agreement with our findings, Sato et al. showed that in healthy women, MK7 was found to be more bioavailable than MK4 [54]. In addition, Shea and Holden reviewed the relation between dietary vitamin K intake and vascular calcification and they concluded that dietary menaquinone intake may be more likely to protect against vascular calcification than phylloquinone [55]. In this respect, MK7, due to its improved bioavailability compared to other dietary menaquinones, could be more effective than the other forms of vitamin K. Indeed, Nakamura et al. suggested that the optimal dose of MK4 to decrease inactive osteocalcin should be 600 µg/day or more [56], which is much higher than the required dose for MK7 (180 µg/day) [57]. In relation to the different activities observed, it must be noted that both bioavailability and the antioxidant properties reported for quinones are strongly dependent on the length of the side chain and the model system used [58,59,60]. It is therefore plausible that different biological activities are related to the higher lipophilicity of MK7 compared to other K vitamins. Moreover, in the inner mitochondrial membrane, size specific interference cannot be excluded among quinones of different length which may explain the contradictory result relative to MK4 reported here.

## 5. Conclusions

In conclusion, the present study reveals that supplementation with K1 and MK4 was not effective in preventing changes induced by CSE-exposure in endothelial cells, such as the alteration in oxidative status and mitochondrial dysfunction which lead to viability loss, caspase-1 activation and early cellular senescence. On the contrary, optimistic results were obtained with the use of QH and MK7 which effectively counteracted viability loss, oxidative stress, inflammation and CSE-induced premature aging. Therefore, QH and MK7 are promising molecules that could have important implications in the prevention and treatment of endothelial dysfunction, although further investigations are required to understand their mechanisms of action, especially with regards to MK7.

## Supplementary Materials

The following are available online at https://www.mdpi.com/2076-3921/9/10/1008/s1, Figure S1: Representative chromatogram obtained by HPLC system for K vitamins and CoQ_10_; Figure S2: Representative flow cytometric graphs; Figure S3: Cells with high content of ROS after 24 h treatment of young HUVECs with increasing doses of CSE; Figure S4: Effect of quinones supplementation on viability and oxidative stress in young HUVECs exposed to CSE (0.425 puffs/mL for 24 h).

## References

[1] Roth G.A., Johnson C., Abajobir A., Abd-Allah F., Abera S.F., Abyu G., Ahmed M., Aksut B., Alam T., Alam K. (2017). Global, regional, and national burden of cardiovascular diseases for 10 causes, 1990 to 2015. J. Am. Coll. Cardiol..

[2] Ezzati M., Obermeyer Z., Tzoulaki I., Mayosi B.M., Elliott P., Leon D.A. (2015). Contributions of risk factor trends and medical care to cardiovascular mortality trends. Nat. Rev. Cardiol..

[3] Rodgman A., Perfetti T.A. (2013). The Chemical Components of Tobacco and Tobacco Smoke.

[4] Farhat N., Thorin-Trescases N., Voghel G., Villeneuve L., Mamarbachi M., Perrault L.P., Carrier M., Thorin E. (2008). Stress-induced senescence predominates in endothelial cells isolated from atherosclerotic chronic smokers. Can. J. Physiol. Pharmacol..

[5] Li D., Mehta J.L. (2009). Intracellular signaling of LOX-1 in endothelial cell apoptosis. Circ. Res..

[6] Frey R.S., Ushio-Fukai M., Malik A.B. (2009). NADPH oxidase-dependent signaling in endothelial cells: Role in physiology and pathology. Antioxid. Redox Signal..

[7] Orosz Z., Csiszar A., Labinskyy N., Smith K., Kaminski P.M., Ferdinandy P., Wolin M.S., Rivera A., Ungvari Z. (2007). Cigarette smoke-induced proinflammatory alterations in the endothelial phenotype: Role of NAD(P)H oxidase activation. Am. J. Physiol. Circ. Physiol..

[8] Yang G.Y., Zhang C.L., Liu X.C., Qian G., Deng D.Q. (2013). Effects of cigarette smoke extracts on the growth and senescence of skin fibroblasts in vitro. Int. J. Biol. Sci..

[9] Wu Q., Jiang D., Matsuda J.L., Ternyak K., Zhang B., Chu H.W. (2016). Cigarette smoke induces human airway epithelial senescence via growth differentiation factor 15 production. Am. J. Respir. Cell Mol. Biol..

[10] Liu A., Wu J., Li A., Bi W., Liu T., Cao L., Liu Y., Dong L. (2016). The inhibitory mechanism of Cordyceps sinensis on cigarette smoke extract-induced senescence in human bronchial epithelial cells. Int. J. Chronic Obstr. Pulm. Dis..

[11] He Z., Chen Y., Hou C., He W., Chen P. (2017). Cigarette smoke extract changes expression of endothelial nitric oxide synthase (eNOS) and p16(INK4a) and is related to endothelial progenitor cell dysfunction. Med. Sci. Monit..

[12] Bentinger M., Brismar K., Dallner G. (2007). The antioxidant role of coenzyme Q. Mitochondrion.

[13] Failla M.L., Chitchumroonchokchai C., Aoki F. (2014). Increased bioavailability of ubiquinol compared to that of ubiquinone is due to more efficient micellarization during digestion and greater GSH-dependent uptake and basolateral secretion by Caco-2 cells. J. Agric. Food Chem..

[14] Tsai K.L., Huang Y.H., Kao C.L., Yang D.M., Lee H.C., Chou H.Y., Chen Y.C., Chiou G.Y., Chen L.H., Yang Y.P. (2012). A novel mechanism of coenzyme Q10 protects against human endothelial cells from oxidative stress-induced injury by modulating NO-related pathways. J. Nutr. Biochem..

[15] Schmelzer C., Lorenz G., Rimbach G., Doring F. (2009). In vitro effects of the reduced form of coenzyme Q (10) on secretion levels of TNF-α and chemokines in response to LPS in the human monocytic cell line THP-1. J. Clin. Biochem. Nutr..

[16] Lee B.J., Tseng Y.F., Yen C.H., Lin P.T. (2013). Effects of coenzyme Q10 supplementation (300 mg/day) on antioxidation and anti-inflammation in coronary artery disease patients during statins therapy: A randomized, placebo-controlled trial. Nutr. J..

[17] Olivieri F., Lazzarini R., Babini L., Prattichizzo F., Rippo M.R., Tiano L., Di Nuzzo S., Graciotti L., Festa R., Bruge F. (2013). Anti-inflammatory effect of ubiquinol-10 on young and senescent endothelial cells via miR-146a modulation. Free Radic. Biol. Med..

[18] Huo J., Xu Z., Hosoe K., Kubo H., Miyahara H., Dai J., Mori M., Sawashita J., Higuchi K. (2018). Coenzyme Q10 prevents senescence and dysfunction caused by oxidative stress in vascular endothelial cells. Oxidative Med. Cell. Longev..

[19] Niklowitz P., Onur S., Fischer A., Laudes M., Palussen M., Menke T., Doring F. (2016). Coenzyme Q10 serum concentration and redox status in European adults: Influence of age, sex, and lipoprotein concentration. J. Clin. Biochem. Nutr..

[20] Schottlaender L.V., Bettencourt C., Kiely A.P., Chalasani A., Neergheen V., Holton J.L., Hargreaves I., Houlden H. (2016). Coenzyme Q10 levels are decreased in the cerebellum of multiple-system atrophy patients. PLoS ONE.

[21] Shearer M.J., Newman P. (2014). Recent trends in the metabolism and cell biology of vitamin K with special reference to vitamin K cycling and MK-4 biosynthesis. J. Lipid Res..

[22] Ohsaki Y., Shirakawa H., Miura A., Giriwono P.E., Sato S., Ohashi A., Iribe M., Goto T., Komai M. (2010). Vitamin K suppresses the lipopolysaccharide-induced expression of inflammatory cytokines in cultured macrophage-like cells via the inhibition of the activation of nuclear factor kB through the repression of IKKα/β phosphorylation. J. Nutr. Biochem..

[23] Lee H., Park J.R., Kim E.J., Kim W.J., Hong S.H., Park S.M., Yang S.R. (2016). Cigarette smoke-mediated oxidative stress induces apoptosis via the MAPKs/STAT1 pathway in mouse lung fibroblasts. Toxicol. Lett..

[24] Carp H., Janoff A. (1978). Possible mechanisms of emphysema in smokers. In vitro suppression of serum elastase-inhibitory capacity by fresh cigarette smoke and its prevention by antioxidants. Am. Rev. Respir. Dis..

[25] Giannubilo S.R., Orlando P., Silvestri S., Cirilli I., Marcheggiani F., Ciavattini A., Tiano L. (2018). CoQ10 supplementation in patients undergoing IVF-ET: The relationship with follicular fluid content and oocyte Maturity. Antioxidants.

[26] Damiani E., Brugè F., Cirilli I., Marcheggiani F., Olivieri F., Armeni T., Cianfruglia L., Giuliani A., Orlando P., Tiano L. (2018). Modulation of oxidative status by normoxia and hypoxia on cultures of human dermal fibroblasts: How does it affect cell aging?. Oxidative Med. Cell. Longev..

[27] Giuliani A., Cirilli I., Prattichizzo F., Mensà E., Fulgenzi G., Sabbatinelli J., Graciotti L., Olivieri F., Procopio A.D., Tiano L. (2018). The mitomiR/Bcl-2 axis affects mitochondrial function and autophagic vacuole formation in senescent endothelial cells. Aging.

[28] Carnevali S., Petruzzelli S., Longoni B., Vanacore R., Barale R., Cipollini M., Scatena F., Paggiaro P., Celi A., Giuntini C. (2003). Cigarette smoke extract induces oxidative stress and apoptosis in human lung fibroblasts. Am. J. Physiol. Cell. Mol. Physiol..

[29] Csiszar A., Labinskyy N., Podlutsky A., Kaminski P.M., Wolin M.S., Zhang C., Mukhopadhyay P., Pacher P., Hu F., de Cabo R. (2008). Vasoprotective effects of resveratrol and SIRT1: Attenuation of cigarette smoke-induced oxidative stress and proinflammatory phenotypic alterations. Am. J. Physiol. Circ. Physiol..

[30] Csordas A., Kreutmayer S., Ploner C., Braun P.R., Karlas A., Backovic A., Wick G., Bernhard D. (2011). Cigarette smoke extract induces prolonged endoplasmic reticulum stress and autophagic cell death in human umbilical vein endothelial cells. Cardiovasc. Res..

[31] Messner B., Frotschnig S., Steinacher-Nigisch A., Winter B., Eichmair E., Gebetsberger J., Schwaiger S., Ploner C., Laufer G., Bernhard D. (2012). Apoptosis and necrosis: Two different outcomes of cigarette smoke condensate-induced endothelial cell death. Cell Death Dis..

[32] Faux S.P., Tai T., Thorne D., Xu Y., Breheny D., Gaca M. (2009). The role of oxidative stress in the biological responses of lung epithelial cells to cigarette smoke. Biomarkers.

[33] Slebos D.J., Ryter S.W., van der Toorn M., Liu F., Guo F., Baty C.J., Karlsson J.M., Watkins S.C., Kim H.P., Wang X. (2007). Mitochondrial localization and function of heme oxygenase-1 in cigarette smoke-induced cell death. Am. J. Respir. Cell Mol. Biol..

[34] Csiszar A., Podlutsky A., Wolin M.S., Losonczy G., Pacher P., Ungvari Z. (2009). Oxidative stress and accelerated vascular aging: Implications for cigarette smoking. Front. Biosci..

[35] Martinon F., Mayor A., Tschopp J. (2009). The inflammasomes: Guardians of the body. Annu. Rev. Immunol..

[36] Kurz D.J., Decary S., Hong Y., Erusalimsky J.D. (2000). Senescence-associated (β)-galactosidase reflects an increase in lysosomal mass during replicative ageing of human endothelial cells. J. Cell Sci..

[37] Hossain M., Mazzone P., Tierney W., Cucullo L. (2011). In vitro assessment of tobacco smoke toxicity at the BBB: Do antioxidant supplements have a protective role?. BMC Neurosci..

[38] Kaisar M.A., Prasad S., Cucullo L. (2015). Protecting the BBB endothelium against cigarette smoke-induced oxidative stress using popular antioxidants: Are they really beneficial?. Brain Res..

[39] Willems B.A., Vermeer C., Reutelingsperger C.P., Schurgers L.J. (2014). The realm of vitamin K dependent proteins: Shifting from coagulation toward calcification. Mol. Nutr. Food Res..

[40] Van der Meer J.H., van der Poll T., Van’t Veer C. (2014). TAM receptors, Gas6, and protein S: Roles in inflammation and hemostasis. Blood.

[41] Gairola C.G., Howatt D.A., Daugherty A. (2010). Dietary coenzyme Q10 does not protect against cigarette smoke-augmented atherosclerosis in apoE-deficient mice. Free Radic. Biol. Med..

[42] Tian G., Sawashita J., Kubo H., Nishio S.Y., Hashimoto S., Suzuki N., Yoshimura H., Tsuruoka M., Wang Y., Liu Y. (2014). Ubiquinol-10 supplementation activates mitochondria functions to decelerate senescence in senescence-accelerated mice. Antioxid. Redox Signal..

[43] Nowicka B., Kruk J. (2010). Occurrence, biosynthesis and function of isoprenoid quinones. Biochim. Biophys. Acta BBA Bioenerg..

[44] Schwalfenberg G.K. (2017). Vitamins K1 and K2: The emerging group of vitamins required for human health. J. Nutr. Metab..

[45] Ivanova D., Zhelev Z., Getso P., Nikolova B., Aoki I., Higashi T., Bakalova R. (2018). Vitamin K: Redox-modulation, prevention of mitochondrial dysfunction and anticancer effect. Redox Biol..

[46] Vos M., Esposito G., Edirisinghe J.N., Vilain S., Haddad D.M., Slabbaert J.R., Van Meensel S., Schaap O., De Strooper B., Meganathan R. (2012). Vitamin K2 is a mitochondrial electron carrier that rescues pink1 deficiency. Science.

[47] Yang Y.M., Liu G.T. (2004). Damaging effect of cigarette smoke extract on primary cultured human umbilical vein endothelial cells and its mechanism. Biomed. Environ. Sci..

[48] Shamas-Din A., Satsoura D., Khan O., Zhu W., Leber B., Fradin C., Andrews D.W. (2014). Multiple partners can kiss-and-run: Bax transfers between multiple membranes and permeabilizes those primed by tBid. Cell Death Dis..

[49] Wu K., Luan G., Xu Y., Shen S., Qian S., Zhu Z., Zhang X., Yin S., Ye J. (2020). Cigarette smoke extract increases mitochondrial membrane permeability through activation of adenine nucleotide translocator (ANT) in lung epithelial cells. Biochem. Biophys. Res. Commun..

[50] Syed F.M., Hahn H.S., Odley A., Guo Y., Vallejo J.G., Lynch R.A., Mann D.L., Bolli R., Dorn G.W. (2005). Proapoptotic effects of caspase-1/interleukin-converting enzyme dominate in myocardial ischemia. Circ. Res..

[51] Müller T., Hengstermann A. (2012). Nrf2: Friend and foe in preventing cigarette smoking-dependent lung disease. Chem. Res. Toxicol..

[52] Giebe S., Cockcroft N., Hewitt K., Brux M., Hofmann A., Morawietz H., Brunssen C. (2017). Cigarette smoke extract counteracts atheroprotective effects of high laminar flow on endothelial function. Redox Biol..

[53] Fratta Pasini A., Albiero A., Stranieri C., Cominacini M., Pasini A., Mozzini C., Vallerio P., Cominacini L., Garbin U. (2012). Serum oxidative stress-induced repression of Nrf2 and GSH depletion: A mechanism potentially involved in endothelial dysfunction of young smokers. PLoS ONE.

[54] Sato T., Schurgers L.J., Uenishi K. (2012). Comparison of menaquinone-4 and menaquinone-7 bioavailability in healthy women. Nutr. J..

[55] Shea M.K., Holden R.M. (2012). Vitamin K status and vascular calcification: Evidence from observational and clinical studies. Adv. Nutr..

[56] Nakamura E., Aoki M., Watanabe F., Kamimura A. (2014). Low-dose menaquinone-4 improves γ-carboxylation of osteocalcin in young males: A non-placebo-controlled dose-response study. Nutr. J..

[57] Knapen M.H., Drummen N.E., Smit E., Vermeer C., Theuwissen E. (2013). Three-year low-dose menaquinone-7 supplementation helps decrease bone loss in healthy postmenopausal women. Osteoporos. Int..

[58] Halder M., Petsophonsakul P., Akbulut A.C., Pavlic A., Bohan F., Anderson E., Maresz K., Kramann R., Schurgers L. (2019). Vitamin K: Double bonds beyond coagulation insights into differences between Vitamin K1 and K2 in health and disease. Int. J. Mol. Sci..

[59] Roche Y., Peretti P., Bernard S. (2006). Influence of the chain length of ubiquinones on their interaction with DPPC in mixed monolayers. Biochim. Biophys. Acta BBA Biomembr..

[60] Okada K., Kainou T., Matsuda H., Kawamukai M. (1998). Biological significance of the side chain length of ubiquinone in *Saccharomyces cerevisiae*. FEBS Lett..

