# Supplementary material for: The Protective Role of Bioactive Quinones in Stress-induced Senescence Phenotype of Endothelial Cells Exposed to Cigarette Smoke Extract
Source: Antioxidants (Basel). 2020 Oct 16;9(10):1008. doi: 10.3390/antiox9101008 (PMC7602940; doi:10.3390/antiox9101008)
Supplement: Supplementary file 1 [file antioxidants-09-01008-s001.pdf]

Supplementary Materials

The Protective Role of Bioactive Quinones in Stress-induced Senescence Phenotype of Endothelial Cells Exposed to Cigarette Smoke Extract

Ilenia Cirilli [1], Patrick Orlando [1], Fabio Marcheggiani [1], Phiwayinkosi V. Dludla [1,2], Sonia Silvestri [1,*], Elisabetta Damiani [1 *] and Luca Tiano [1]

1 Department of Life and Environmental Sciences, Polytechnic University of Marche, Ancona, Italy; i.cirilli@pm.univpm.it (I.C.); p.orlando@univpm.it (P.O.); f.marcheggiani@univpm.it (F.M.); l.tiano@staff.univpm.it (L.T.)

2 Biomedical Research and Innovation Platform, South African Medical Research Council, Tygerberg, South Africa; phiwayinkosi.dludla@mrc.ac.za (P.V.D.)

* Correspondence: e.damiani@univpm.it (E.D.); Tel.: +39-071-220-4135 (E.D.); s.silvestri@univpm.it (S.S.); Tel.: +39-071-220-4394 (S.S.)

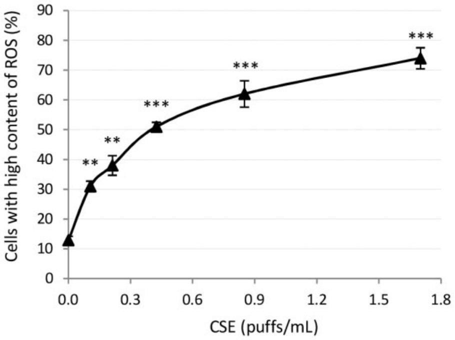

Figure S1. Cells with high content of ROS after 24 h treatment of young HUVECs with increasing doses of CSE. Significance was calculated with respect to young untreated cells (0 puffs/mL CSE). * p≤0.05, ** p≤0.01, *** p≤0.001.

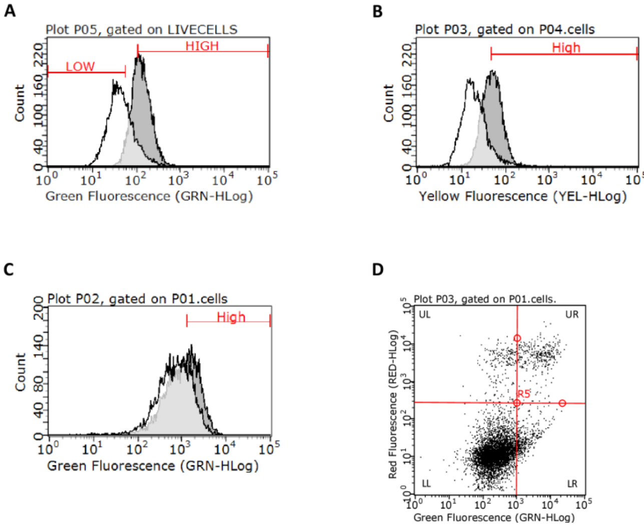

Figure S2. Representative flow cytometry graphs. Fluorescence distribution of (A) total ROS, (B) mitochondrial ROS, (C) mPTP opening, (D) caspase-1: LL: live cells; LR: live cells with activated caspase-1; UR: apoptotic or death cells with activated caspase-1; UL: apoptotic or death cells without activated caspase-1.

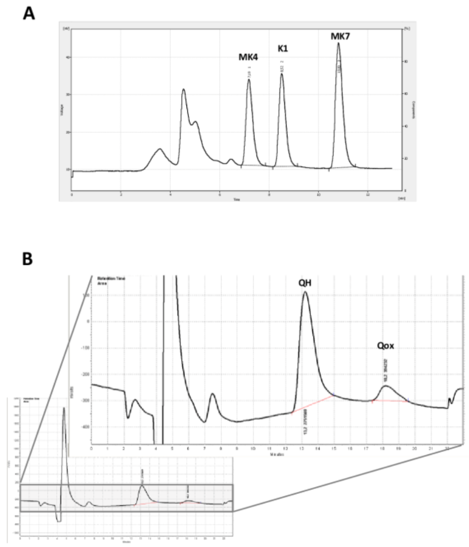

Figure S3. Representative chromatogram obtained by HPLC system for (A) K vitamins and (B) CoQ_{10}.

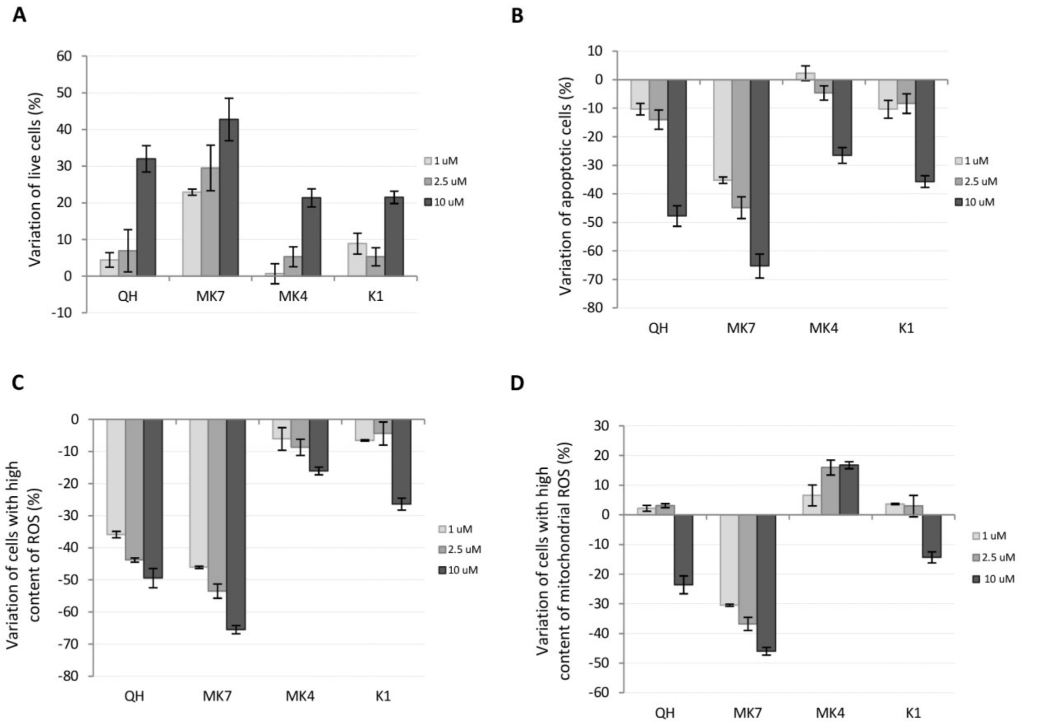

Figure S4. Effect of quinones supplementation on viability and oxidative stress in young HUVECs exposed to CSE (0.425 puffs/mL for 24 h). Cells were supplemented with different concentrations of quinones for 24 h followed by replacement with CSE medium containing the same quinones and incubated for a further 24 h. Variation of the percentage of: (A) live cells, (B) apoptotic cells, (C) cells with high ROS content, (D) cells with high mitochondrial ROS content.
